# Click-chemistry-based protocol to quantitatively assess fatty acid uptake by *Mycobacterium tuberculosis* in axenic culture and inside mouse macrophages

**DOI:** 10.1016/j.xpro.2023.102062

**Published:** 2023-01-24

**Authors:** Thomas Laval, Caroline Demangel

**Affiliations:** 1 Immunobiology and Therapy Unit, Institut Pasteur, INSERM U1224, Paris, France; 2 Université Paris Cité, Paris, France

**Keywords:** Cell Biology, Cell Culture, Flow Cytometry/Mass Cytometry, Immunology, Metabolism, Microbiology, Microscopy, Molecular/Chemical Probes

## Abstract

*Mycobacterium tuberculosis* (Mtb) hijacks host-derived fatty acids (FAs) to sustain its intracellular growth inside host cells. Here, we present a click-chemistry-based protocol to assess FA import by Mtb in axenic culture or inside mouse macrophages. We describe the use of alkyne analogs of natural FAs as an alternative to structurally altered fluorescent derivatives or hazardous radiolabeled FAs. We also detail quantitative analyses of FA uptake at single bacterial or host cell level by flow cytometry and confocal fluorescence microscopy.

For complete details on the use and execution of this protocol, please refer to Laval et al. (2021).^1^

## Highlights

• Protocol to quantify fatty acid uptake by mycobacteria in culture or within macrophages
• An alternative to protocols using radioactive or fluorescent fatty acids
• Using commercially available fatty acid analogs with minor structural alteration
• Compatible with single-cell analyses by flow cytometry and fluorescence microscopy

## Abstract (editor highlights)

Publisher’s note: Undertaking any experimental protocol requires adherence to local institutional guidelines for laboratory safety and ethics.

## Graphical abstract

**Figure:**
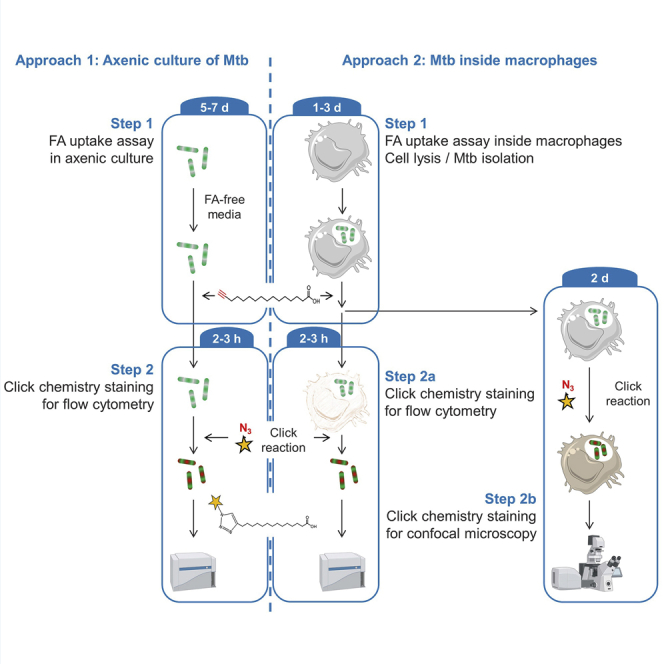

## Before you begin

As an intracellular pathogen, *Mycobacterium tuberculosis* (Mtb) relies on host-derived lipids including fatty acids (FAs) for survival and growth *in vivo*.^2^ Mtb’s ability to import FAs in axenic cultures or inside macrophages was previously studied using radiolabeled or fluorophore-conjugated FAs, two approaches with advantages and disadvantages.^3^^,^^4^^,^^5^ While radiolabeled FAs are structurally identical to the native compounds, they require an appropriate infrastructure and are not compatible with single cell analyses. Conversely, fluorescent derivatives of FAs allow such analyses but the covalent addition of relatively large fluorophores greatly alters their structure and physicochemical properties. This may impact their import and use by bacteria and eukaryotic cells, as reported for a commonly-used fluorescent derivative of glucose.^6^^,^^7^^,^^8^

Here, we describe a protocol adapted from the one previously published by Nazarova and colleagues and using a BODIPY-conjugated FA.^9^ This new version uses commercially available alkyne-FAs that are structurally close to the native FAs and can be detected by a highly specific and versatile ‘click’ reaction (copper(I)-catalyzed alkyne-azide cycloaddition) with a picolyl azide-tagged fluorophore.^10^ The steps below describe the various applications of this protocol, using the example of Mtb strain H37Rv grown in axenic culture or internalized by murine bone marrow-derived macrophages (BMDMs). We have also used this protocol successfully with *M. bovis* BCG and human PMA-differentiated THP-1 macrophages.

All media and solutions must be prepared before experiments (see recipes in the materials and equipment section below). They can be stored as indicated in the footnotes of the recipes.

**CRITICAL:** It is important to consider the age, sex, and background of the mice used in this protocol. We have used 7- to 12-week-old male C57BL/6J mice housed under specific pathogen-free conditions with food and water *ad libitum*.

### Institutional permissions

All animal procedures were performed in agreement with European and French guidelines (Directive 86/609/CEE and Decree 87–848 of 19 October 1987). The study received the approval by the Institut Pasteur Safety Committee (Protocol 11.245).

### Preparation of L929-conditioned medium

**Timing: 7 days**

1. Grow L929 cells from frozen stocks, and split the culture to plate 1 × 10^6^ cells in 100 mL of complete DMEM in a TC-treated 150-cm^2^ culture flask.
2. Incubate cells for 7 days at 37°C and 5% CO_2_.
3. Harvest the cell culture medium.
  a. Centrifuge the pooled L929-conditioned medium at 200 × *g* for 5 min at 22°C.
  b. Pool all supernatants and filter them through a sterile 0.22 μm filter.
  c. Aliquot in 50 mL tubes and store at −20°C for up to a year.

To take into account variations in the concentration of M-CSF secreted by L929, a quality check of each new batch of conditioned medium (ability to promote BMDM differentiation, see section below) is recommended.

### Preparation of bone marrow-derived macrophages (BMDMs)

**Timing: 7 days**

4. For microscopy experiments, prepare a 24-well plate containing sterile coverslips.
  a. Autoclave glass coverslips and tweezers with a dry cycle.
  b. In a biosafety cabinet, place coverslips in pure ethanol in a Petri dish.
  c. Crook a sterile, beveled needle with autoclaved tweezers. Using tweezers and the crooked needle, transfer coverslips in the wells of the plate.
  d. Let the ethanol evaporate for 1 h in the biosafety cabinet.
  e. Wash the wells containing the coverslips once with 1 mL of sterile 1× DPBS before seeding the cells.
5. Euthanize a C57BL/6J mouse using the approved methods of primary and secondary euthanasia at your institution. Use 70% ethanol to clean the exterior of the animal.
6. In a biosafety cabinet, dissect out femur and tibia bones and place them in 1× DPBS as described previously by Toda and colleagues.^11^
7. Soak the bones in 70% ethanol for 1 min, transfer in new sterile 1× DPBS, cut off extremities and flush them with 1× DPBS using a syringe and a 25G needle as described.^11^
8. Homogenize the cell suspension by pipetting up and down, and pass it through a 70-μm cell strainer on top of a sterile 50 mL conical tube.
9. To count the isolated hematopoietic cells, dilute 10 μL of cell suspension in 90 μL of Türk’s solution, and use a Malassez counting chamber.
10. Seed 7 × 10^6^ cells in 10 mL of BMDM differentiation medium per 100 mm TC-treated cell culture dish, or 2 × 10^5^ cells in 500 μL medium per well of a 24-well plate containing autoclaved coverslips.
11. Culture bone marrow cells for 6 days, with medium change on day 3.
12. After 6 days of differentiation, replace BMDM differentiation medium with BMDM culture medium and incubate another 24 h at 37°C and 5% CO_2_.

***Note:*** Using this method, 60–80 million bone marrow cells can reproducibly be isolated from one 7–12 week-old C57BL/6J male mouse.

***Note:*** The quality of BMDM differentiation can be assessed by flow cytometry as described previously by Toda and colleagues.^11^ A 90% rate of CD11b- and F4/80-positive cells is expected with this protocol.

***Note:*** Alternative protocols have been described for the generation of BMDMs. We have not tested if the outcome of the assays described below is different with these alternative protocols. Any alterations of BMDM lipid metabolism (specifically their FA import capacity) could have significant effects on the assay outcome.

## Key resources table

**Table:** 

REAGENT or RESOURCE	SOURCE	IDENTIFIER
**Bacterial and virus strains**		

*Mycobacterium tuberculosis* H37Rv	Gift from L. Majlessi, Institut Pasteur, Paris	N/A
*Mycobacterium bovis* BCG	Gift from R. Brosch, Institut Pasteur, Paris	N/A
GFP-expressing *M. tuberculosis* H37Rv WT	Laval et al.^1^	N/A
GFP-expressing *M. tuberculosis* H37Rv *Δmce1D*	Laval et al.^1^	N/A
GFP-expressing *M. tuberculosis* H37Rv *Δmce1D Comp*	Laval et al.^1^	N/A

**Chemicals, peptides, and recombinant proteins**		

Natural fatty acids	Cayman Chemical	Cat#10006627; 90260; 90010; 90150; 90110; 90310
Alkyne fatty acids	Cayman Chemical	Cat#13266; 10541; 9002078; 10538; 16704; 16689
ProLong Diamond Antifade Mountant	Thermo Fisher Scientific	Cat#P36961
DAPI stain	Sigma-Aldrich	Cat#D9542
16% Paraformaldehyde (PFA)	Electron Microscopy Sciences	Cat#15710
1× DPBS	Thermo Fisher Scientific	Cat#14190144
Bovine serum albumin (BSA) fraction V	PAN BIOTECH	Cat#P06-1403500
Glycerol	Sigma-Aldrich	Cat#G6279
Fatty acid-free BSA	Sigma-Aldrich	Cat#A9205
Tween 80	Sigma-Aldrich	Cat#P4780
Triton X-100	Sigma-Aldrich	Cat#X100
Tyloxapol	Sigma-Aldrich	Cat#T0307
Sodium chloride	Sigma-Aldrich	Cat#S9888
Dextrose	Sigma-Aldrich	Cat#D9434
Sucrose	Sigma-Aldrich	Cat#S0389
EGTA	Sigma-Aldrich	Cat#E3889
HEPES	Sigma-Aldrich	Cat#H3375
Kanamycin	Sigma-Aldrich	Cat#K0254
Streptomycin	Sigma-Aldrich	Cat#S9137
Zeocin	Invivogen	Cat#ant-zn-1
Dulbecco’s modified Eagle’s medium (DMEM) GlutaMAX™	Thermo Fisher Scientific	Cat#10566016
Penicillin-Streptomycin (100×)	Thermo Fisher Scientific	Cat#15140122
Heat-inactivated fetal bovine serum (FBS)	Dominique Dutcher	Cat#S1810500
Türk’s solution	Sigma-Aldrich	Cat#93770
Middlebrook 7H9 broth base	Sigma-Aldrich	Cat#M0178
BD BBL™ Middlebrook OADC enrichment	Thermo Fisher Scientific	Cat#211886
Gelatin from cold water fish skin	Sigma-Aldrich	Cat#G7765

**Critical commercial assays**		

Click-iT^TM^ Plus Alexa Fluor™ 647 Picolyl Azide Toolkit	Thermo Fisher Scientific	Cat#C10643

**Experimental models: Cell lines**		

*Mus musculus*: L929 cell line, Passage 5–15	ATCC	Cat#CCL-1

**Experimental models: Organisms/strains**		

*Mus musculus*: C57BL/6J, adult (7–12 weeks), male	Charles River Laboratories	Cat# JAX: 000664; RRID : IMSR_JAX :000664

**Software and algorithms**		

Zen Imaging software	Zeiss	RRID: SCR_013672
Icy opensource platform	de Chaumont et al.^12^	http://www.icy.bioimageanalysis.org RRID: SCR_010587
FlowJo software	Becton Dickinson	https://www.flowjo.com/ RRID: SCR_008520
GraphPad Prism	GraphPad	https://graphpad.com RRID: SCR_015807

**Other**		

TPP 100 mm cell culture dish	Thermo Fisher Scientific	Cat#93100
TPP 24-well plate	Thermo Fisher Scientific	Cat#92024
12 mm-diameter coverslips	VWR Scientific	Cat# MENZCB00120RAC20
BD Luer-Lok^TM^ 1-mL syringes	Becton Dickinson	Cat#309628
25G sterile hypodermic needles	Becton Dickinson	Cat#300600
27G sterile hypodermic needles	Becton Dickinson	Cat#300635
70 μm cell strainers	Becton Dickinson	Cat#352350
FACS tubes with 35 μm cell strainer	Becton Dickinson	Cat#352235
GentleMACS™ M tubes	Miltenyi Biotec	Cat#130093236; RRID: SCR_020269
GentleMACS™ Dissociator	Miltenyi Biotec	Cat#130093235; RRID: SCR_020269
CytoFLEX Flow Cytometer	Beckman Coulter	RRID: SCR_019627
LSM 700 confocal microscope	Zeiss	RRID: SCR_017377

## Materials and equipment

**Table:** 7H9 broth

Reagent	Final concentration	Amount
7H9 Broth Base	N/A	1.68 g
Glycerol	0.2%	0.72 mL
Water	N/A	Up to 358 mL
**Total**	**N/A**	**358 mL**

7H9 broth is autoclaved at 121°C for 10 min and stored at 20°C–25°C for 3 months maximum.

**Table:** 7H9-OADC-Tylox medium

Reagent	Final concentration	Amount
7H9 Broth	N/A	358 mL
OADC enrichment	10%	40 mL
Tyloxapol 10%	0.05%	2 mL
**Total**	**N/A**	**400 mL**

Tyloxapol is prediluted in water at 50°C–60°C, filter-sterilized (0.22 μm) and stored at 20°C–25°C. Prepared medium is stored at 4°C for up to 2 months.

**Table:** 7H9-AD medium

Reagent	Final concentration	Amount
7H9 Broth	N/A	45 mL
Dextrose 100 g/L	2 g/L	1 mL
Sodium chloride 34 g/L	0.85 g/L	1.25 mL
Fatty acid-free BSA 10%	0.5%	2.5 mL
Tyloxapol 10%	0.05%	0.25 mL
**Total**	**N/A**	**50 mL**

Dextrose and sodium chloride are prediluted in water, filter-sterilized (0.22 μm) and stored at 4°C. Prepared medium is stored at 4°C for up to 2 months.

**Table:** Pre-conjugated FA-BSA

Reagent	Final concentration	Amount
Natural or alkyne fatty acid	600 μM	variable
Fatty acid-free BSA 10%	300 μM (2%)	variable
**Total**	**N/A**	variable

Pure fatty acids are stored in ethanol at −20°C for up to 3 months. All FAs supplied by Cayman Chemical are delivered in ethanol at different concentrations, except for natural palmitic acid powder that has to be dissolved at 60 mM in ethanol with sonication. For conjugation, the FA-BSA solution is incubated for 20 min at 37°C before use in bacteria or cell culture medium.

**Table:** 4% PFA

Reagent	Final concentration	Amount
Paraformaldehyde (PFA) 16%	4%	10 mL
Sterile 1× DPBS	N/A	30 mL
**Total**	**4%**	**40 mL**

4% PFA is aliquoted and stored at −20°C for up to 3 months. Thawed aliquots stored at 4°C are used within a week.

**Table:** Homogenization Buffer

Reagent	Final concentration	Amount
Sucrose	85.5 g/L	85.5 g
EGTA	190 mg/L	190 mg
HEPES	4.76 g/L	4.76 g
Gelatin 45%	0.0225%	0.5 mL
Water	N/A	Up to 1 L
**Total**	**N/A**	**1 L**

pH of the Homogenization Buffer is adjusted at 7 before filter-sterilization (0.22 μm) and storage at 4°C for 2 months maximum.

**Table:** 10% Tween 80

Reagent	Final concentration	Amount
Tween 80	10%	1 mL
Water	N/A	9 mL
**Total**	**10%**	**10 mL**

10% Tween 80 is filter-sterilized (0.22 μm) and stored at 20°C–25°C for 3 months maximum.

**Table:** Wash Buffer

Reagent	Final concentration	Amount
DPBS (1×, sterile)	N/A	49 mL
Fatty acid-free BSA 10%	0.1%	0.5 mL
Triton X-100 10%	0.1%	0.5 mL
**Total**	**N/A**	**50 mL**

Triton X-100 is prediluted in water, filter-sterilized (0.22 μm) and stored at 20°C–25°C. Wash Buffer is stored at 4°C and used within 2 weeks.

**Table:** Permeabilization Buffer

Reagent	Final concentration	Amount
DPBS (1×, sterile)	N/A	9.5 mL
Triton X-100 10%	0.5%	0.5 mL
**Total**	**N/A**	**10 mL**

Permeabilization Buffer is made fresh from prediluted X-100 and used within one day.

**Table:** Click-iT^TM^ Plus reaction cocktail

Reagent	Final concentration	Amount
1× Click-iT reaction buffer	N/A	261 μL
Alexa Fluor 647 PCA (500 μM)	5 μM	3 μL
CuSO_4_-copper protectant pre-mix	N/A	6 μL
1× Click-iT buffer additive	N/A	30 μL
**Total**	**N/A**	**300 μL**

1× buffers are prepared as per the manufacturer’s instructions (https://www.thermofisher.com/order/catalog/product/C10643). For the pre-mix, 2 μL of copper protectant are diluted in 8 μL of CuSO_4_. The reaction cocktail is used within 15 min of preparation.

**Table:** Complete DMEM

Reagent	Final concentration	Amount
DMEM GlutaMAX	N/A	445 mL
Heat-inactivated FBS	10%	50 mL
Penicillin-Streptomycin	1×	5 mL
**Total**	**N/A**	**500 mL**

FBS is heat-inactivated at 56°C for 30 min, filter-sterilized (0.22 μm) and stored at 4°C for 1 month maximum. Complete DMEM is stored at 4°C for up to 2 months.

**Table:** BMDM differentiation medium

Reagent	Final concentration	Amount
DMEM GlutaMAX	N/A	370 mL
Fetal Bovine Serum (FBS)	10%	50 mL
Penicillin-Streptomycin	1×	5 mL
L929-conditioned medium	15%	75 mL
**Total**	**N/A**	**500 mL**

BMDM differentiation medium is stored at 4°C for up to 2 months.

**Table:** BMDM culture medium

Reagent	Final concentration	Amount
DMEM GlutaMAX	N/A	425 mL
Fetal Bovine Serum (FBS)	10%	50 mL
L929-conditioned medium	5%	25 mL
**Total**	**N/A**	**500 mL**

BMDM culture medium is stored at 4°C for up to 2 months.

## Step-by-step method details

### Assay of fatty acid uptake by Mtb in axenic cultures

**Timing: 10–14 days**

This protocol was adapted from that previously reported for the uptake of radiolabeled FAs by Mtb grown in axenic culture.^3^^,^^4^ The variation presented below uses flow cytometry to assess quantitatively the uptake of alkyne-FAs at the single-bacterial level *in vitro*.

1. Grow GFP-expressing Mtb strains for 5–7 days to an OD_600_ of 0.8–1 in standing culture flask at 37°C in 7H9-OADC-Tylox medium containing selection antibiotics.

***Note:****gfp* was expressed by our Mtb strains under the control of the pBlaF∗ promoter.^1^

***Note:*** Antibiotics were used at the final concentrations of 40 μg/mL (kanamycin), 25 μg/mL (streptomycin), or 50 μg/mL (zeocin).

2. Subculture Mtb for FA uptake assay.
  a. Centrifuge (3,000 × *g* for 5 min) 1 mL of bacterial culture.
  b. Discard supernatant and wash the bacterial pellet once with 1 mL of 7H9 broth.
  c. Resuspend the washed bacteria in 5 mL of FA-free 7H9-AD medium.
  d. Grow for 5–7 days at 37°C in standing culture flask until bacteria reach an OD_600_ of 0.6–0.8.

**CRITICAL:** When comparing FA uptake by different bacterial strains, making sure that bacterial cultures have similar OD_600_ is crucial since the expression of Mtb lipid transporters depends on the growth phase.^13^^,^^14^

3. Alkyne FA uptake:
  a. To make a heat-killed negative control, incubate an aliquot of the culture for 15 min at 80°C.
  b. Let it cool down to 22°C–25°C before incubation with alkyne FAs.
    ***Note:*** An alternative is to kill bacteria by treatment with 4% PFA for 30 min at 22°C–25°C, before centrifugation (9,000 × *g* for 5 min) and resuspension in alkyne-FA-supplemented 7H9-AD.
  c. Dilute 8.34 μL of pre-conjugated alkyne FA-BSA in 992 μL of 7H9-AD to have a final FA concentration of 5 μM.
    ***Note:*** For competition assays, add pre-conjugated natural FA-BSA at a final concentration of 5–50 μM.
  d. Centrifuge (3,000 × *g*) bacteria for 5 min at 22°C–25°C.
  e. Resuspend bacteria to OD_600_ of 0.1 in FA-supplemented medium.
  f. Incubate for 1 h at 37°C.
4. Centrifuge (3,000 × *g*) bacteria for 5 min at 4°C.
5. Wash bacterial pellets once in ice-cold 7H9-AD medium.
6. Wash bacterial pellets twice in ice-cold Wash Buffer.

***Note:*** These washing steps were optimized to minimize the amount of FAs that are bound to the mycobacterial envelope without being internalized, using heat-killed Mtb as a metabolically inert control.

7. Fix bacteria by incubation in 4% PFA for 1 h at 22°C–25°C.
8. Proceed to click chemistry staining and flow cytometry analysis.

### Assay of fatty acid uptake by Mtb inside BMDMs

**Timing: 1–3 days**

This modified version of the protocol published by Nazarova and colleagues^9^ allows to test the capacity of intracellular Mtb to import alkyne-FAs by flow cytometry (in 100 mm cell culture dish, with 10 mL of culture medium) or confocal microscopy (on glass coverslip in 24-well plate, with 500 μL of culture medium).

9. Grow Mtb in 7H9-OADC-Tylox medium until late log phase (OD_600_ = 0.6–0.8).
10. Differentiate bone marrow cells into a monolayer of BMDMs as described above, and incubate for 24 h in antibiotic-free BMDM culture medium before Mtb infection.
11. Prepare Mtb for macrophage infection:
  a. Centrifuge (3,000 × *g*) 30 mL of a bacterial culture for 5 min.
  b. Wash the pellet twice in 30 mL of 1× DPBS, and resuspend in 5 mL of sterile 1× DPBS.
  c. Dissociate bacteria in M-tubes with the “RNA01.01” program of the gentleMACS™ dissociator as previously described.^15^
  d. Pass bacteria through a 25G (20 times) and a 27G needle (one time) with a syringe and let it stand for 10 min to exclude remaining clumps.
  e. Transfer the suspension into a new tube, and measure the OD_600_ of the suspension.
  f. Dilute Mtb at OD_600_ = 0.01–0.02 in antibiotic-free BMDM culture medium to infect macrophages.

***Note:*** Since BMDMs are not replated after differentiation in this protocol, we use an approximative multiplicity of infection (MOI) of 2 bacilli per cell, assuming that an OD_600_ of 1 corresponds to 1.5 × 10^8^ bacterial/mL and that the BMDM monolayer corresponds to 7 × 10^6^ cells in 100 mm dish and 2 × 10^5^ cells/well in 24-well plate.

12. Replace the culture medium with 5 mL (100 mm dish) or 200 μL (24-well plate) of the diluted Mtb.
13. Incubate for 3 h at 37°C and 5% CO_2_.
14. Wash infected cells 3 times with warm DMEM to remove extracellular bacteria, and add fresh BMDM culture medium.

***Note:*** DMEM should never be pipetted directly onto adherent cells, specifically at this washing step, as it could detach them.

15. Incubate cells for 1 or 3 days at 37°C and 5% CO_2_.
16. Alkyne FA pulse-chase:
  a. Dilute pre-conjugated alkyne FA-BSA in BMDM culture medium at a final FA concentration of 5 μM, with the same dilution factor as above (step 3b).
  b. Replace cell supernatant with equivalent volume of alkyne FA-supplemented BMDM culture medium.
    ***Note:*** As a negative control for the click staining, you can prepare infected BMDMs that are incubated with BMDM culture medium without alkyne FA.
  c. Incubate for 1 h at 37°C and 5% CO_2_.
  d. Replace cell supernatant with an equivalent volume of DMEM GlutaMAX supplemented with 10% FBS.
  e. Incubate for 1 h at 37°C and 5% CO_2_.
    **CRITICAL:** For confocal microscopy analysis, proceed to click chemistry staining as described in the next section. For flow cytometry analysis of FA uptake by Mtb, intracellular bacteria have to be isolated from infected BMDMs prior to staining.

17. Isolate intracellular bacteria from infected cells in 100 mm cell culture dish:
  a. Wash infected BMDMs twice with 5 mL of ice-cold Homogenization Buffer.
  b. Add 5 mL of ice-cold Homogenization Buffer and incubate for 10–15 min at 4°C.
  c. Scrape BMDMs with a cell scraper and transfer cells into a 15 mL conical tube.
  d. Centrifuge (500 × *g*) cells for 10 min at 4°C.
  e. Resuspend the pellet in 1 mL of Homogenization Buffer.
  f. Pass the cells at least 20–25 times through a 25G needle, plus once through a 27G needle using a 1 mL syringe. Keep the cells at 4°C throughout the isolation protocol.
    ***Note:*** Cell lysis can be verified by cell observation in a counting slide with a microscope.
  g. Add 4 mL of Homogenization Buffer.
  h. Centrifuge (200 × *g*) for 10 min at 4°C.
  i. Carefully transfer the supernatant containing most cytoplasmic material from lysed cells to a new tube.
  j. Add to the supernatant 50 μL of 10% Tween 80 (final concentration of 0.1%).
  k. Pipet up and down vigorously until foam formation.
    **CRITICAL:** Thorough mixing at this stage and subsequent washing are crucial to dissolve cellular FA-containing membranes associated with bacteria.
  l. Incubate for 15 min at 4°C.
  m. Centrifuge (1,500 × *g*) for 15 min at 4°C.
  n. Resuspend well the bacterial pellet in 1 mL of ice-cold 1× DPBS with 0.05% Tyloxapol.
  o. Centrifuge (3,000 × *g*) for 5 min at 4°C.
  p. Wash with 1 mL of cold 1× DPBS-0.05% Tyloxapol.
  q. Centrifuge (3,000 × *g*) for 5 min at 4°C.
  r. Wash with 1 mL of cold Wash Buffer.
  s. Centrifuge (3,000 × *g*) for 5 min at 4°C.
  t. Resuspend bacteria in 200 μL of 4% PFA.
  u. Incubate 16–20 h at 4°C before staining for flow cytometry analysis.

### Click chemistry staining for analysis by confocal microscopy

**Timing: 2 days**

This staining method allows quantification of alkyne-FAs in Mtb of infected BMDMs by confocal microscopy, using the Click-iT^TM^ Plus Alexa Fluor™ 647 Picolyl Azide Toolkit.

18. After the FA uptake assay described above, wash twice with ice-cold 1× DPBS the infected BMDMs cultured on coverslips in 24-well plates.
19. Add 300 μL of 4% PFA and incubate for 1 h at 22°C–25°C.
20. Wash the cells twice with 500 μL of 1× DPBS.
21. Add 300 μL of Permeabilization Buffer.
22. Incubate for 10 min at 22°C–25°C.
23. Wash the cells twice with 500 μL of 3% BSA in 1× DPBS.
24. Add 300 μL of Click-iT^TM^ Plus reaction cocktail.

***Note:*** The copper:protectant ratio (see materials and equipment section) in the cocktail was optimized for this staining to minimize the deleterious effects of copper on GFP fluorescence while conserving a maximal reaction efficiency (as indicated by the clicked FA signal in bacteria incubated with alkyne FAs), as per the manufacturer’s indications (https://www.thermofisher.com/order/catalog/product/C10643).

***Note:*** Although we optimized this click reaction to be compatible with GFP-expressing Mtb, we noticed that copper has much less deleterious effects on the fluorescence of RFP-expressing Mtb. Therefore, the amount of copper may be adjusted when using strains expressing other fluorescent proteins.

**Figure 1:**
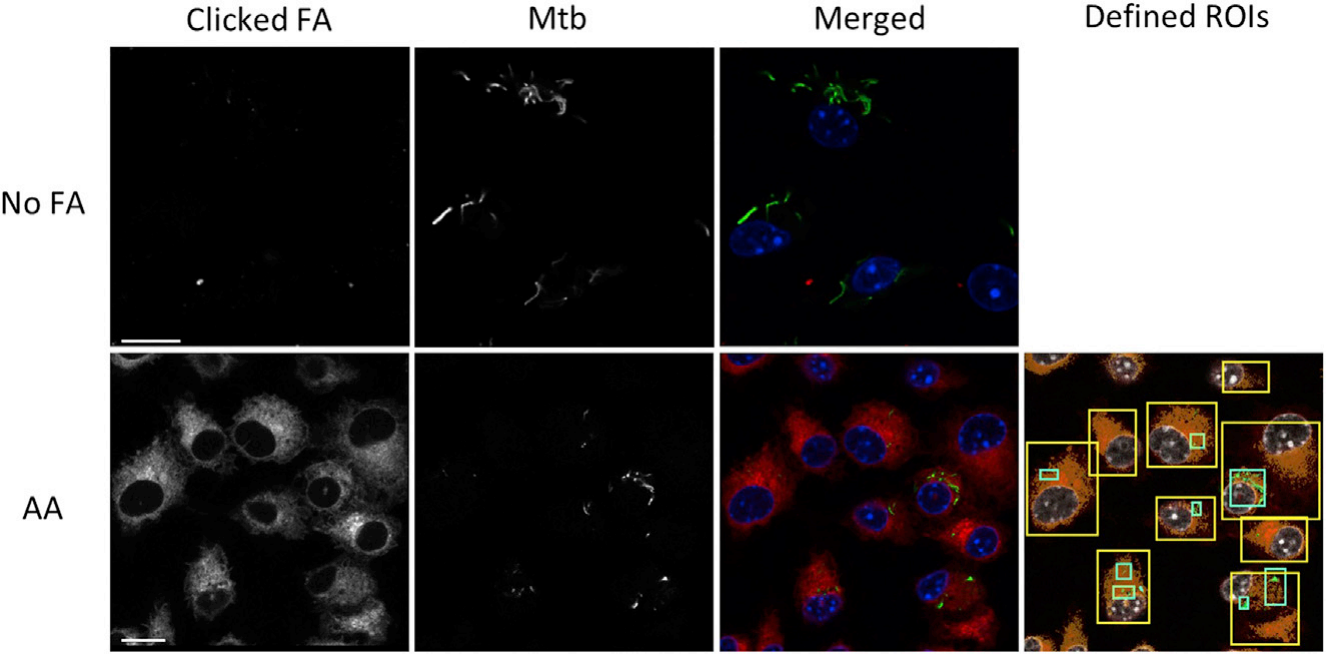
Defining Mtb and BMDM ROIs using HK-Means Distribution of alkyne-arachidonic acid (AA) in Mtb-infected BMDMs at 24 h post infection, as shown on a confocal image (green = GFP-expressing Mtb, red = clicked FA, blue = DAPI). For quantification of the FA signal, ROIs (shown in azure blue and yellow ochre for Mtb and BMDMs, respectively) were defined using the HK-Means plugin. Bar scale = 10 μm.
  b. Measure the mean fluorescence intensity in defined ROIs.

25. Incubate for 30 min at 22°C–25°C.
26. Wash the cells twice with 500 μL of 3% BSA in 1× DPBS.
27. Add 300 μL of DAPI (1 μg/mL) in 1× DPBS.
28. Incubate for 5 min at 22°C–25°C.
29. Wash the cells 3 times with 500 μL of 1× DPBS, and add the same volume of 1× DPBS.
30. Using tweezers and a crooked, beveled needle, get coverslips from the wells, dab the coverslip edge on a Kim wipe and flip them over onto a drop of Prolong Diamond Antifade Mountant on a glass slide.
31. Let it cure for 24 h at 22°C–25°C before sealing with nail polish, and storage at 4°C.
32. Image cells using a 63×/1.4 oil objective on a LSM 700 inverted confocal microscope and Zen Imaging software, or equivalent.
33. For each acquisition, set up the z-scan spanning so that most intracellular bacteria are fully imaged.
34. For quantification of FA uptake, perform all acquisitions using the same settings.
  a. Define Mtb and BMDM regions of interest (ROIs) by the GFP and clicked FA signal, respectively, using the HK-Means plugin^16^ of the Icy opensource platform^12^ (see Figure 1).

### Click chemistry staining for analysis by flow cytometry

**Timing: 2–3 h**

This protocol uses the Click-iT^TM^ Plus Alexa Fluor™ 647 Picolyl Azide Toolkit to assess and quantify alkyne-FA import by Mtb from axenic culture or infected macrophages.

35. Centrifuge (9,000 × *g*) fixed Mtb (from axenic culture or isolated from infected BMDMs) for 5 min at 22°C–25°C.

***Note:*** This speed is necessary to pellet fixed bacteria in a buffer devoid of detergent (otherwise, bacteria tend to stick to the walls of plastic tubes).

36. Add 700 μL of Permeabilization Buffer.
37. Incubate for 15 min at 22°C–25°C.
38. Add 300 μL of 10% BSA.
39. Centrifuge (5,000 × *g*) for 5 min at 22°C–25°C.
40. Resuspend bacteria in 300 μL of Click-iT^TM^ Plus reaction cocktail.

***Note:*** See notes in the previous section related to the reaction cocktail.

**Figure 2:**
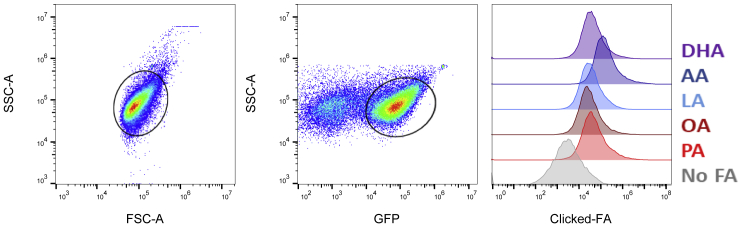
Gating strategy for the flow cytometry analysis of FA uptake by Mtb A GFP-expressing strain of Mtb was isolated from infected BMDMs at 24 h post infection after a pulse-chase exposure to alkyne palmitic (PA), oleic (OA), linoleic (LA), arachidonic (AA) or docosahexaenoic acid (DHA). Imported alkyne FAs were stained by click chemistry of isolated Mtb and analyzed by flow cytometry based on GFP and clicked FA fluorescence. Data were extracted from Laval et al.^1^ with permission (https://creativecommons.org/licenses/by/4.0/).
49. Analyze the AF647 fluorescence signal in the APC channel, using GFP-expressing bacteria isolated from infected macrophages not treated with alkyne-FAs as negative control.

***Note:*** There is not requirement for compensation when using GFP and AF647, since they have negligible spectrum overlap.

***Note:*** Care should be taken to assess FA uptake by host macrophages during infection, as it determines FA bioavailability for intracellular Mtb.^1^ FA uptake by infected macrophages can easily be assessed in parallel by confocal microscopy as described above, or by staining unlysed cells (following the Click-IT^TM^ toolkit manufacturer’s instruction (https://www.thermofisher.com/order/catalog/product/C10643) and flow cytometry analysis.

***Note:*** For assays in axenic cultures, we do not completely eliminate bacterial clumps that may form throughout the protocol, but simply use the 35-μm cell strainer to avoid large clump formation. However, when a very heterogenous population is observed in flow cytometry analyses (indicative of large clumps), alkyne-FA signals are normalized by FSC values for each event using the “Derive Parameters” function in FlowJo.

41. Incubate for 30 min at 22°C–25°C.
42. Add 700 μL of cold Wash Buffer.
43. Centrifuge (5,000 × *g*) for 5 min at 4°C.
44. Wash bacteria twice with 1 mL of cold Wash Buffer.
45. Centrifuge (5,000 × *g*) for 5 min at 4°C.
46. Resuspend in 7H9 plus 0.85 g/L NaCl and 0.05% Tyloxapol.
47. Read samples on a flow cytometer with regular homogenization by pipetting and passage through a 35-μm cell strainer to avoid large clump formation.
48. Select events as indicated in the gating strategy (Figure 2). Exclude small debris by selecting GFP-positive events in the FITC channel.

## Expected outcomes

### FA uptake in axenic culture

**Figure 3:**
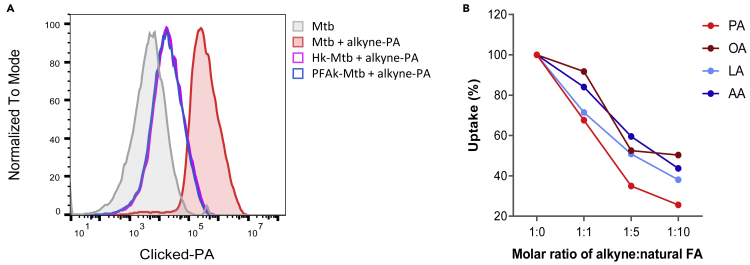
Validation of alkyne-FAs as FA surrogates to assess FA uptake by Mtb in axenic culture (A) Uptake of alkyne-palmitic acid (PA) by live, heat-killed (Hk-Mtb) or paraformaldehyde-killed (PFAk-Mtb) Mtb, as assessed by click chemistry staining of imported alkyne-PA and flow cytometry analysis. (B) Relative uptake of alkyne-PA, - oleic acid (OA), - linoleic acid (LA), and -arachidonic acid (AA) by Mtb WT in the presence of increasing amounts of natural PA, OA, LA, or AA. Data shown are representative of at least two independent experiments.

Using this approach, we confirmed that the uptake of alkyne-FAs is severely impaired in Mtb strains deficient for Mce1, the only specific FA transport system characterized to date in Mtb.^17^ Importantly, we compared FA uptake by live versus heat- or PFA-killed Mtb. As shown in Figure 3A, both killing methods strikingly reduced FA uptake by Mtb, confirming that our assay largely measures active import of FAs. Of note, in killed bacteria we could detect a residual FA signal that is likely corresponding to passive diffusion of alkyne FAs.

To validate that the alkyne modification did not significantly alter the mechanism and efficiency of FA import by Mtb, we performed competition assays with increasing doses of natural FAs added to a fixed dose of the alkyne FA counterpart. As expected, the uptake of all alkyne-FAs was efficiently competed by addition of their natural counterpart (Figure 3B).

### FA uptake inside macrophages

**Figure 4:**
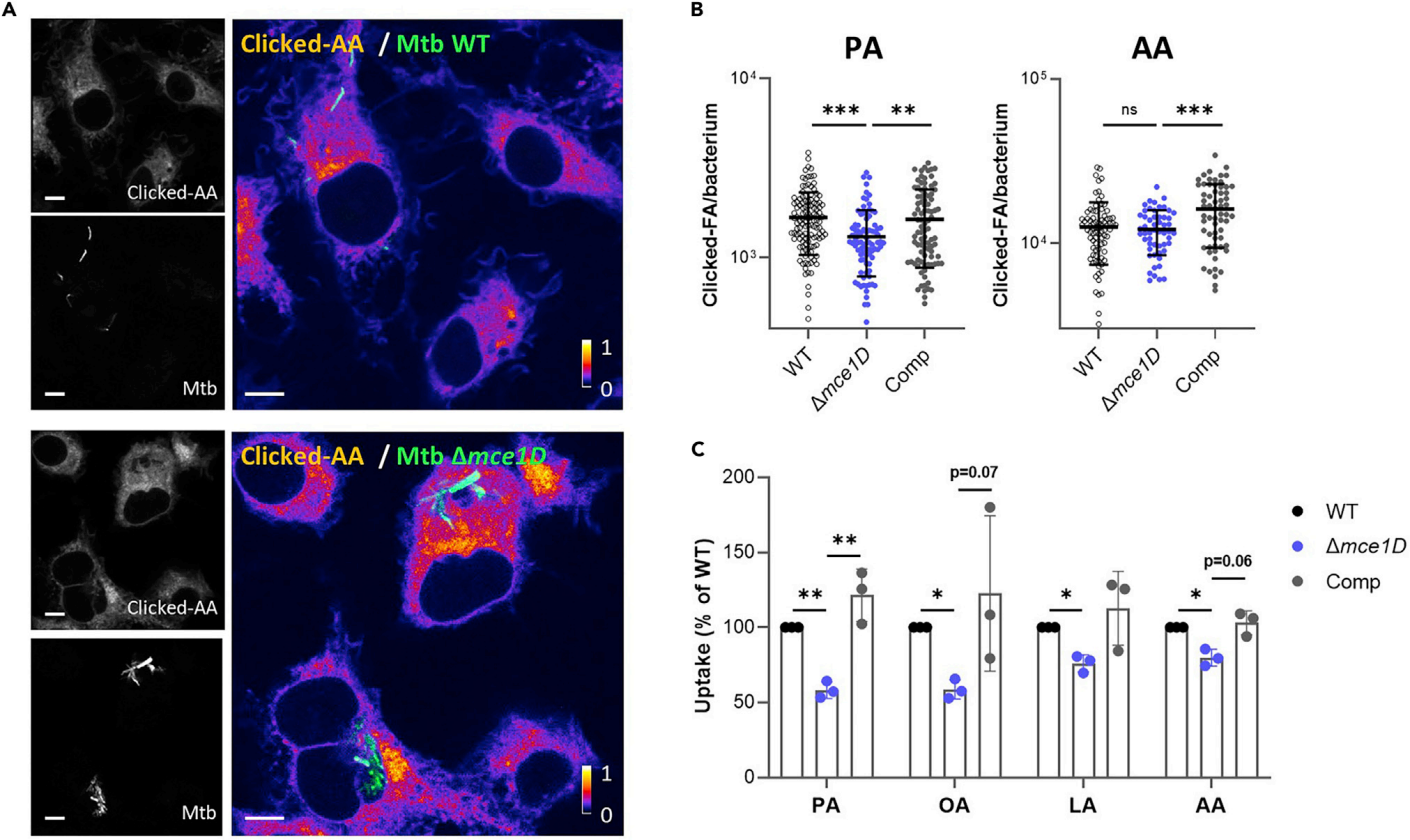
Measurement of Mce1-dependent FA uptake by Mtb inside macrophages (A) Distribution of alkyne-AA in Mtb-infected BMDMs at 24 h post infection, as shown on representative confocal images (green = GFP-expressing Mtb, log scale colormap = clicked AA). Color bar indicates the relative range of pixel intensity (white = high, purple = low, from 0 arbitrary unit to 1). Bar scale = 5 μm. (B) Quantification of the alkyne-FA signal in intracellular Mtb using confocal images of BMDMs infected for 24 h with different GFP-expressing Mtb strains. Bars show means ± SD, n > 54. ns, not significant, ∗∗p < 0.01, ∗∗∗p < 0.001 in a one-way ANOVA with Dunnett post-hoc multiple comparison tests. (C) Uptake of alkyne-FAs by Mtb *Δmce1D* and its complemented counterpart (Comp), recovered from BMDMs infected for 24 h, relative to Mtb WT, as analyzed by flow cytometry. Data are means ± SD from three independent experiments, ∗p < 0.05, ∗∗p < 0.01, paired t-tests. Data were adapted from Laval et al.^1^ with permission (https://creativecommons.org/licenses/by/4.0/).

We were able to detect a significant defect in the import of several alkyne-FAs in Mce1-deficient Mtb (*Δmce1D*) using both confocal microscopy (Figures 4A and 4B) and flow cytometry (Figure 4C). The magnitude of this defect, compared to both wildtype and complemented strains, was consistent with those previously-reported with BODIPY-PA.^17^

## Limitations

A limitation of this protocol is the need of sample fixation and use of high copper amounts for detection, which prevent imaging/sorting of live infected cells. At present, such techniques are only accessible using fluorophore-conjugated FAs, as described by Nazarova and colleagues in a screen of Mtb mutants deficient in FA import during macrophage infection.^17^ A copper-free click reaction may allow to adapt our technique to the study of clickable derivatives by real-time live cell imaging.^18^^,^^19^^,^^20^

## Troubleshooting

### Problem 1

The difference of FA signal in Mtb is small between the positive (wildtype, live Mtb) and negative (killed or Mce1-deficient Mtb) controls in flow cytometry analyses.

### Potential solution

For axenic cultures, make sure that all Mtb strains were at the same growth stage. Although we were able to measure significant differences after 1 h of FA uptake and have not tested other timepoints for these comparisons, bigger differences may be observed with longer uptake period. If the problem persists, bacteria should be washed more thoroughly before fixation (assay of fatty acid uptake by Mtb in axenic cultures, steps 5 and 6 & assay of fatty acid uptake by Mtb inside BMDMs, steps 17.k–q).

### Problem 2

The signal of the fluorescent protein expressed by the Mtb strain used is very low in click stained compared to unstained samples.

### Potential solution

This signal depends on the copper sensitivity and expression levels (controlled by the strength of the promoter used) of the fluorescent protein in your Mtb strains. The copper:protectant ratio in the Click-iT^TM^ Plus reaction cocktail (materials and equipment (optional), click chemistry staining for analysis by confocal microscopy, step 24) may have to be adjusted to limit the deleterious effects of copper on the fluorescent protein.

### Problem 3

There is minimal competition between an alkyne-FA and its natural counterpart for uptake by Mtb in axenic culture.

### Potential solution

This may be caused by a lack of stability or solubility of the compounds. Make sure you keep the ethanol stock solutions of FAs as cold and away from light sources as possible while preparing the pre-conjugated FA-BSA (materials and equipment (optional)). In case of doubt, replace the stock solution by an unopened one (unsaturated FAs are particularly sensitive to oxidation). To ensure good solubility in the culture medium, mix thoroughly FAs in pre-warmed FA-free BSA and incubate for a minimum of 20 min at 37°C (this time can be increased, but without exceeding 1 h to limit the oxidation of unsaturated FAs).

## Resource availability

### Lead contact

Further information and requests for resources should be directed to Caroline Demangel (demangel@pasteur.fr).

### Materials availability

The *M. tuberculosis* strains used in this protocol may be made available upon request.

## Data Availability

This study did not generate/analyze datasets or code.
